# Current state of electronic problems lists in primary care: a rapid scoping review

**DOI:** 10.1093/fampra/cmag036

**Published:** 2026-06-11

**Authors:** Rajesh Nair, Ian Bekker, Alexander Singer, Francis Lau

**Affiliations:** School of Health Information Science HSD Building, Room A202, 3800 Finnerty Road, Victoria, BC, Canada V8P 5C2; Department of Family Medicine, Island Health, Victoria, Canada V8R 1J8; Department of Family Medicine, University of Manitoba, Manitoba Primary Care Research Network, 770 Bannatyne Avenue, Winnipeg, MA, Canada R3E 0W3; School of Health Information Science HSD Building, Room A202, 3800 Finnerty Road, Victoria, BC, Canada V8P 5C2

**Keywords:** electronic problem list, digital problem list, problem-oriented medical record, primary care

## Abstract

**Background:**

Electronic problem lists (PLs) are central to the problem-oriented medical record and increasingly underpin clinical decision support (CDS), interoperability, and emerging artificial intelligence applications in primary care. However, persistent concerns regarding PL accuracy, completeness, and governance limit their clinical value.

**Objective:**

To update the foundational framework proposed by Hodge and Narus by synthesizing contemporary evidence on electronic PLs in primary care and primary care-relevant settings.

**Methods:**

We conducted a rapid scoping review following Joanna Briggs Institute guidance and PRISMA-ScR reporting standards. MEDLINE (Ovid) was searched for peer-reviewed studies published between March 2016 and May 2026. Eligible studies examined digital PLs or extractable PL-related practices relevant to primary care. Findings were synthesized using a hybrid inductive-deductive approach and mapped to the seven Hodge and Narus themes.

**Results:**

A total of 103 studies were included. Across settings, PLs were widely recognized as foundational for longitudinal care, safety, and coordination. However, incomplete, outdated, or inconsistently maintained PLs remained common. Persistent challenges included unclear ownership, workflow misalignment, and variability in what constitutes a “problem.” Emerging technologies—including CDS, natural language processing, and machine learning—were increasingly used to support PL generation, curation, reconciliation, and organization, with greatest benefit when embedded within clinician-led workflows.

**Conclusion:**

Despite technological advances, the clinical value of electronic PLs in primary care continues to depend on governance, workflow integration, and shared accountability. Updating the Hodge and Narus framework highlights the need for sociotechnical approaches to PL improvement as PLs become integral to CDS and artificial intelligence-enabled care.

Key messages

- Electronic PLs are a core clinical tool in primary care but are frequently incomplete, outdated, or inconsistently maintained.
- Persistent challenges—including unclear ownership, poor workflow integration, and disagreement about what belongs on the PL—continue despite advances in EHR technology.
- Increasing use of PL for CDS, interoperability, and AI-enabled tools amplifies the clinical risks associated with poor data quality.
- Automation and AI can assist with PL curation and reconciliation but are most effective when embedded within clinician-led workflows and supported by clear governance.
- Sustainable improvement in PL quality requires explicit role delineation, team-based responsibility, and leadership within primary care settings.

## Introduction

Electronic (or digital) problem lists (PLs) within electronic health records (EHR) systems continue to fall short of Lawrence Weed's original vision for the problem-oriented medical record and its intended clinical impact [1, 2]. Weed conceptualized the medical record as comprising four integrated components: (i) a comprehensive patient database, (ii) an explicit list of the patient's problems, (iii) plans of action linked to each problem, and (iv) progress notes organized around those problems [1–4].

In both paper and digital charting, the PL is intended to provide a concise, accurate, and up-to-date representation of a patient's active diseases, conditions, and clinically significant health issues [3, 5]. The transition from paper to EHRs introduced clear theoretical advantages, including the ability to maintain a shared, longitudinal PL accessible to multiple members of the care team and updated in real-time [3, 5]. When accurate and well-maintained, PLs have been associated with improvements in patient safety, clinical workflow efficiency, clinical decision-making, and adherence to evidence-based care [3, 5]. Accordingly, PL maintenance is often framed as a core professional responsibility across inpatient and outpatient settings [3, 5].

Primary care providers (PCPs) are uniquely positioned to benefit from high-quality PLs [6]. Patients receiving primary care frequently experience multimorbidity, require longitudinal management, and transition across providers and care settings [6]. In this context, the PL has the potential to function as a shared cognitive workspace that supports synthesis of complex clinical information, care planning, and interdisciplinary communication[7]. Despite this promise, substantial deficiencies persist. Studies consistently demonstrate that between 30% and 50% of clinically relevant chronic conditions are omitted from PLs, while others remain outdated, duplicated, or insufficiently specified [8, 9]. A key contributor to these shortcomings is the lack of standardization in PL implementation, governance, and use across EHR systems [3, 5], limiting realization of their intended clinical value.

In a landmark thematic synthesis, Hodge and Narus systematically analyzed the PL literature and identified seven themes critical to PL success: (i) benefits of using a PL; (ii) the unused PL; (iii) aspects critical to success; (iv) policy; (v) what belongs on the PL; (vi) who should maintain the PL; and (vii) when the PL should be updated [10]. Their work reframed PL quality as a sociotechnical phenomenon, emphasizing that effective PL use depends not only on documentation accuracy, but also on governance clarity, role accountability, workflow integration, and organizational support [10]. This framework remains highly influential and continues to underpin contemporary discussions on PL design and implementation.

Since the publication of Hodge and Narus’ review in 2018, EHR systems and clinical practice environments have undergone substantial evolution. Team-based care models, have expanded, interoperability demands have increased, and PLs are now increasingly leveraged as structured inputs for clinical decision support (CDS), automation, and secondary data use [11–13]. In parallel, advances in artificial intelligence (AI), —including machine learning (ML), natural language processing (NLP), and large language models —have intensified interest in using structured EHR data to support diagnosis, risk stratification, alerting, and workflow augmentation in primary care [14, 15]. Recent evidence suggests that AI applications in community-based primary health care may improve documentation, decision support, and population-level surveillance, while also raising concerns related to data quality, bias, governance, and clinician trust [14, 15]. Critically, the effectiveness and safety of these technologies depend fundamentally on the quality, structure, and governance of PL data, renewing attention to longstanding deficiencies identified in earlier literature.

Despite this rapid evolution, no recent synthesis has systematically mapped contemporary PL research against Hodge and Narus’ original framework, particularly in primary care-relevant contexts. Existing studies tend to focus on isolated outcomes such as completeness, automation accuracy, or clinician perceptions, without situating findings within a comprehensive, theory-informed structure [3, 5]. Moreover, given the pace of technological change, methodological guidance increasingly supports the use of rapid scoping reviews to update foundational syntheses when evidence has expanded but conceptual frameworks remain stable [16].

Accordingly, this rapid scoping review builds directly on the framework developed by Hodge and Narus. A rapid approach was selected to efficiently map recent PL literature, identify areas of convergence and divergence from the original themes, and assess the continued relevance of those themes in the context of modern EHR functionality and emerging AI-enabled applications. The objectives of this rapid scoping review were to: (i) characterize the current state of digital PL research primary care-relevant settings; (ii) map study findings to the seven critical themes identified by Hodge and Narus; and (iii) identify persistent gaps and implications for future PL governance, design, and implementation.

## Materials and methods

This study was conducted as a rapid scoping review, guided by the Joanna Briggs Institute (JBI) methodology for scoping and rapid reviews [16–18]. Reporting followed the preferred reporting items for systematic reviews and meta-analyses extension for scoping reviews (PRISMA-ScR) guidelines [19, 20]. The protocol was developed *a priori* and preregistered on the Open Science Framework (OSF; https://osf.io/3svey).

Consistent with rapid review methodology, several methodological adaptations were applied to balance timeliness with rigor [16–18]. These included restricted to a single bibliographic database, use of a single reviewer for initial screening (R.N.), abbreviated critical appraisal procedures, and structured team-based calibration and validation steps. As the review synthesized published literature only, research ethics approval was not required.

### Research questions

The review was guided by the following research questions:

1. What characteristics of digital PLs facilitate or hinder their use in primary care and primary care-relevant settings?
2. Who is responsible for the creation and maintenance of PLs within these care contexts?
3. How are AI, NLP, and ML approaches used to generate, curate, reconcile, or organize PLs, and how do these approaches impact PL accuracy, completeness, and usability?

### Conceptualization of primary care relevance

Primary care relevance was defined broadly. In addition to traditional primary care settings (family medicine, general practice, community health centers), the review included studies from clinical environments where PL use is conceptually or operationally transferable to primary care. These included ambulatory internal medicine, emergency departments, pediatrics, mental health, hospice, and outpatient specialty clinics. This approach reflects contemporary care delivery, in which PLs are shared across settings and updated longitudinally by multiple clinicians.

### Eligibility criteria

Eligibility was assessed in two stages. First, studies were required to be peer-reviewed publications (quantitative, qualitative, mixed methods, or commentaries with extractable PL-relevant findings), published in English between 11 March 2016 and 15 May 2026. Articles had to examine digital PLs directly or provide clearly extractable insights related to PL structure, content, maintenance, coding practices, workflow integration, governance, or data quality. Commentaries were included only when they described real-world implementations, workflow analyses, or system-level PL practices. Second, studies were required to demonstrate primary care relevance, either through direct study settings (e.g., family medicine or community primary care) or through findings that were conceptually or operationally transferable to primary care contexts, such as PL completeness, coding accuracy, automation, or workflow integration.

Exclusion criteria included dissertations without extractable PL data, conference abstracts, non–peer-reviewed gray literature, non-English publications, and studies published prior to 11 March 2016.

### Search strategy

The literature search was conducted in MEDLINE (Ovid), which served as the single database for this rapid scoping review. The search strategy was designed to capture literature on problem-oriented medical records and digital PLs, while also identifying studies involving automation and advanced computational approaches.

Two concept blocks were combined using Boolean operators. The first captured problem-oriented records and PLs (like Hodge and Narus’ strategy) using both Medical Subject Headings (MeSH) and free-text terms, including “medical records, problem-oriented medical records, problem list, problem-oriented charting, problem summary list, problem-oriented patient record, problem-oriented system,” and “Weed system.” The second concept block captured emerging digital technologies using exploded MeSH terms and free-text keywords for “artificial intelligence, natural language processing, and machine learning.”

The search strategy was developed in collaboration with a clinical informatics librarian and peer-reviewed using the Peer Review of Electronic Search Strategies checklist [21]. The reference list of included studies was also screened to identify any additional eligible articles. The complete search strategy is provided in Supplementary Appendix S1.

### Study selection

All records were imported into Covidence for deduplication and screening. A single primary reviewer (R.N.) conducted title and abstract screening and full-text eligibility assessment. To support reliability, all reviewers (I.B., A.S., and F.L.) participated in calibration exercises at both screening stages using a shared sample of records. Inter-rater reliability was assessed using Cohen's kappa, yielding values of 0.75 (substantial agreement) at the title/abstract stage and 0.59 (moderate agreement) at the full-text stage. Following calibration, discrepancies were resolved through team discussion until consensus was reached.

### Data extraction

Data extraction was performed using a standardized, piloted Microsoft Excel template. Extracted variables included bibliographic information (author, year, country), clinical setting, study design, sample characteristics, PL-related findings, terminology use, workflow considerations, governance models, and AI/NLP applications. The primary reviewer (R.N.) completed initial data extraction for all included studies. Three reviewers (I.B., A.S., and F.L.) independently cross-checked extracted data against the source articles. Discrepancies were resolved through consensus discussion.

### Synthesis and analytic framework

A hybrid inductive-deductive thematic synthesis was undertaken to accommodate the heterogeneity of study designs and clinical contexts [22]. The research questions were developed *a priori* and were not derived from the Hodge and Narus framework. Instead, the framework was applied during the analytic phase to organize and interpret extractable findings. Inductively generated codes were mapped deductively to the seven themes identified by Hodge and Narus: (i) benefits of using PL; (ii) the unused PL; (iii) aspects critical to success; (iv) policy; (v) what belongs on a PL; (vi) who should maintain the PL; and (vii) when should the PL be updated [10]. Findings were synthesized within this sociotechnical framework to support interpretation across diverse clinical and methodological contexts.

### Critical appraisal

Consistent with JBI guidance for scoping and rapid scoping reviews [16–18], no formal design-specific critical appraisal or study exclusion based on methodological quality was performed. However, to support transparency and consistency of interpretation across a heterogeneous evidence base, we adapted selected items from the JBI Critical Appraisal Checklist for Systematic Reviews and Research Syntheses into a pragmatic, review-specific appraisal guide. This modified checklist was used descriptively to document clarity of study aims, appropriateness of methods, transparency of data sources, and completeness of reporting, rather than to generate quality scores or determine study inclusion. The checklist supported team-based calibration and interpretation of findings but did not inform weighting of evidence or exclusion decisions (Supplementary Table S2).

## Results

### Study characteristics

**Figure 1:**
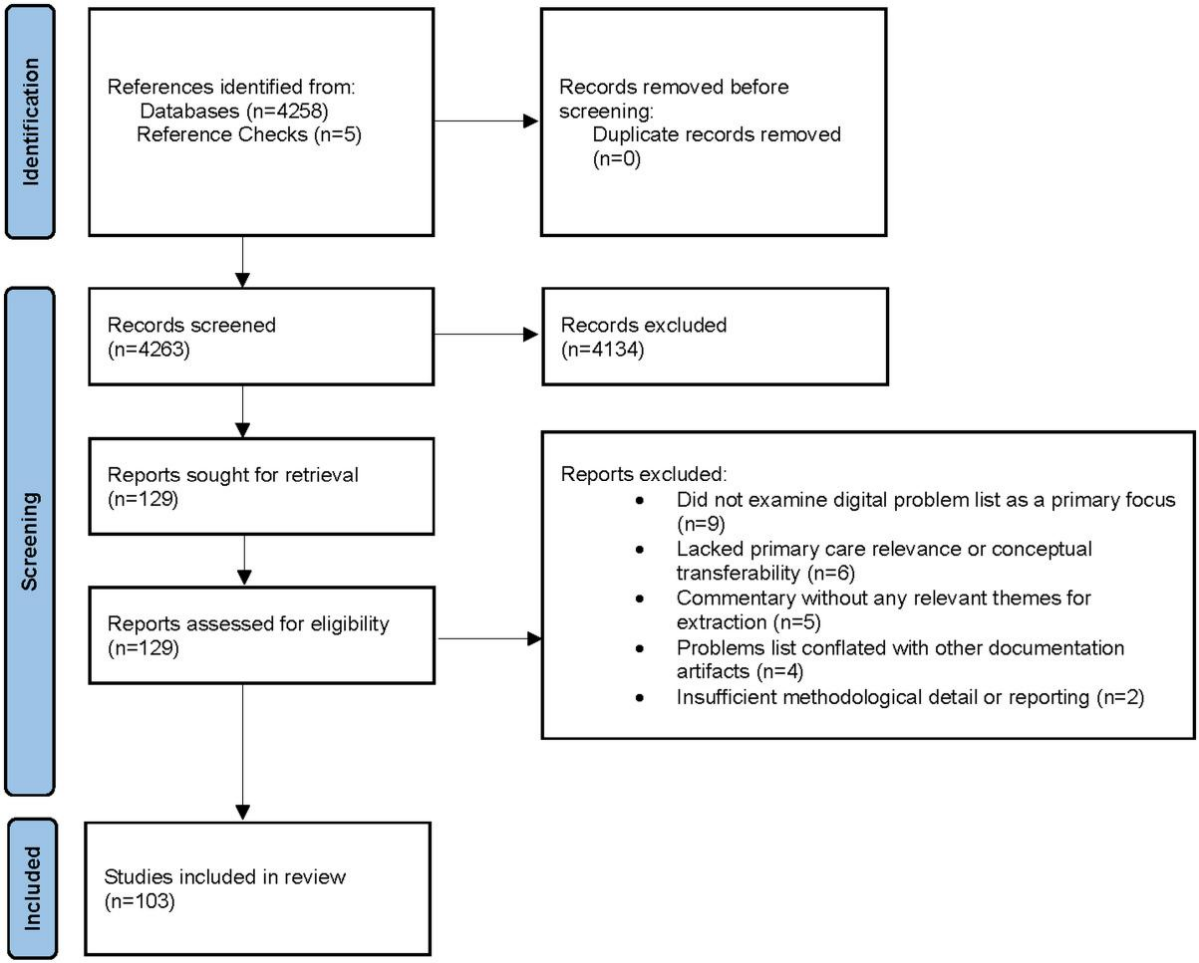
Preferred reporting items for systematic reviews and meta-analyses (PRISMA) flow chart.

A total of 4263 records were imported into Covidence. After excluding 4134 records based on title and abstract screening, 129 full-text articles were assessed for eligibility. Ultimately, 103 studies were included in the final synthesis (Fig. 1) [4, 6, 8, 9, 11–13, 23–118]. Studies were predominantly conducted in North America (*n* = 78; 76%), with Canada accounting for *n* = 14 (14%) and the United States *n* = 64 (62%) (Table 1). European studies represented *n* = 20 (19%), and other regions (Asia, South America, Africa) accounted for *n* = 5 (5%). Overall, nearly half of the evidence base was directly relevant to longitudinal, relationship-based care (*n* = 48; 47%) where PL accuracy and maintenance are foundational to continuity, chronic disease management, and coordination. Observational EHR-based analyses were the most common design (*n* = 44; 43%).

**Table 1:** Characteristics of included articles.

First author, year	Country	Domain/Setting	Study design	Participants	Findings
Altman, 2023 [11]	USA	MC	User-centered design protocol	92 000 visits	A new toolkit in Epic EHR that was customized was successfully adopted across the whole healthcare system.
Borbolla, 2018 [23]	USA	Peds	Qualitative study, critical incident interviews	7 PCPs, 3 care coordinators	Six themes: (1) significant reliance on EHR for situation understanding and organize complexity, (2) need to provide high-level summary making PL central to organize structure, (3) central role of Medical Home model in organizing documentation, (4) integrating info from multiple sources, (5) need for well-organized documentation and meaningful labels for external encounters; (6) need for more effective tools to reconcile meds.
Bowles, 2025 [24]	USA	Home care	Qualitative	91 informants	Inconsistent use of the PL in EHRs was a barrier to identifying sepsis, producing discordance across settings and care.
Brown, 2021 [25]	USA	MC	EHR data warehouse review	546 510 patients	(1) 10% patients had duplicate PL, (2) allergies and family history in PL—move to their specific sections in EHR? (3) policy needed on who should update PL, (4) CDS options—add dx for select meds, resolve/delete dx based on meds, acute conditions auto removed, group conditions by locations ore related problems e.g. problem-oriented charting and/or grouping.
Budde, 2024 [26]	USA	Anesthesia	Quality Improvement	N/A	Epic-based alerts for intubation alerts increased from 9% to 38% postintervention.
Buttafuoco, 2024 [27]	USA	MC	Retrospective cohort	372 patients	Obesity not well documented in PL and results in underutilization of obesity interventions.
Callahan, 2017 [114]	USA	IM	EHR or chart audit	351 IM residents,15 139 patients	Discordance between EHR PLs and chart audits. IM residents poorly document core geriatric conditions. Require further training as part of their core competency requirements.
Campbell, 2020 [113]	USA	MH	EHR data	101 405 patients	PCPs should routinely screen and document cannabis use.
Capito, 2025 [115]	USA	FMC	EHR data	313 patients	Non-interruptive CDS alert increased documented clinical actions; tool include an action to add an exclusion diagnosis to the PL (contradiction) to suppress future alerts, using PL data as part of CDS logic and workflow.
Ceusters, 2018 [28]	USA	MC	EHR database	550 000 patients	PL data should be interpreted with caution when attempting to elucidate comorbidities or ongoing disease. Automated systems can inaccurately infer disease courses over time based on annotation sequences.
Chehal, 2025 [29]	USA	N/A	EHR data	198 160 birthing individuals	Health information systems that flag both early birth outcomes on the PL may help clinicians address known risk factors interconceptionally and prenatally (in a subsequent pregnancy).
Chen, 2016 [30]	Taiwan	MC	EHR data audit and survey-based	5 residents audits, 23 clinicians and non-clinicians	Completeness of PLs were reduced after implementation of a new automated tool. Possible causes include: usability issues, efficacy, lack of incentives, poor leadership support and improper education.
Chen, 2024 [31]	Taiwan	IM	EHR-based dataset	1 206 895 prescriptions	Machine learning-based CDSS tools can improve patient safety by triggering valid clinical alerts.
Cillessen, 2020 [33]	Netherlands	Workshop	Exploratory workshop	132 participants	Some of the key areas discussed include developing patient-centered care, PL maintenance, interoperability, PL linkage with privacy arrangements to different care plans, and a social incentive framework with financial support.
Cillessen, 2021 [32]	Netherlands	MC	EHR database and survey	1765 physician respondents and 193 703 EHR patient records	With patient factors the odds of linking increased with an increase in the number of problems, hospital days, and decreased with an increase in the number of doctors involved in care, medical specialities or the number of notes. With physician factors linking increased with more clinical experience. With physician education and attitudes linking increased with more familiarity in EHR system and belief in the value of POMR. Ultimately leadership needs to be involved to maximize the benefits of linking notes.
Cohen, 2019 [34]	USA	Peds	Sequential explanatory mixed methods	809 physicians, 40 surveys	PL documentation varies between physicians and poor documentation rates can impede effective and safe use of EHRs. Potential mitigation strategies include documentation discussion during regularly scheduled staff meetings and/or updated staff training.
Cyr, 2016 [35]	USA	FM	EHR data	9484 patients	PCPs infrequently document overweight or obese patients on the PL Automation techniques might help improve documentation rates but requires further research.
Daskivich, 2018 [36]	USA	Veterans MC	Retrospective cohort study	1596 male patients	EHR problem list–based comorbidity assessment had poor sensitivity and specificity for detecting major comorbidities, often being inaccurate and leading to incorrect determinations for automated processes. Needs validation prior to any automated process implementation.
Devarakonda, 2017 [37]	USA	IM	Pilot study with EHR data	15 random patient records and 6 physician participants	Cognitive computing systems like machine learning and NLP based models like Watson hold the potential for accurate, PL centered summaries of patient records potentially leading to increased efficiency, better CDS, and improved patient care.
Doghramji, 2018 [38]	USA	MH	Cross-sectional study with EHR data	97 patients	Insomnia is highly prevalent in hospitalized psychiatric patients but poorly identified.
Durojaiye, 2018 [39]	USA	Peds trauma	EHR-based and registry data	517 patient encounters	Most actions in the trauma setting involved addition of items, with updates seldom marked as resolved and rarely deleted.
Flanagan, 2016 [40]	USA	IM	QI	Pre-PLM implementation mean 411 patients/day; post-PLM implementation mean 406 patients/day	Using a multidisciplinary team to implement a PLM was successful in terms of PL usage and documentation improvements. The average number of new problems added to patient's PL doubled after the PLM intervention and increases in PL additions appeared to be relatively sustained to 6 months. The fact that house staff were selective in their addition and inactivation of problems demonstrates the potential for this type of intervention to lead to more accurate and actively maintained PL.
Gammal, 2021 [41]	USA	IM/OP	Commentary	N/A	Integrating pharmacogenomic test results in PLs for use a CDS tool requires specialized training for implementation and maintenance. Specifically, PL documentation and quality needs to be addressed to maximize utilization over time.
Grauer, 2022 [13]	USA	IM/OP	Prospective, observational EHR-data	5 773 495 medication orders	Implementing a clinical decision alert to notify clinicians when ordering medication in the absence of a corresponding indication on the PL improved yield and accuracy of problems.
Harris, 2020 [42]	USA	OP	Retrospective EHR-data	N/A	NLP may improve the utility of PLs commonly found within EHR systems. In this study, NLP PLs were processed with simple rule-based postprocessing.
Heintzman, 2020 [43]	USA	Peds	EHR-data set	37 614 Latino children	Spanish-speaking children were less likely to have the diagnosis of asthma recorded on their PL on the same day it was noted on the chart, but diagnosis was more quickly recorded after the first day.
Hier, 2019 [44]	USA	Community HC	Retrospective EHR-data	4950 PLs over 5-years	Reorganization of the PL into allocation by organ system is feasible using SNOMED CT, as it is a polyhierarchical ontology.
Hodge, 2020 [45]	USA	IM/OP	Survey and EHR-data set	201 PLs	This new model can be used to quantify intervention effects, can be reported in PL studies, and can be used to measure PL changes based on policy, workflow, or system changes.
Horng, 2019 [46]	USA	ED	Prospective EHR-data	180 424 ED visit consecutively	Developed a polyhierarchical ontology containing 692 unique concepts, 2118 synonyms, and 30 613 nonvisible descriptions to correct misspellings and nonstandard terminology. Problem ontology data set was successfully able to capture 96% of a patient's presenting problem with each concept being mapped to SNOMED CT.
Hose, 2019 [47]	USA	Peds	Qualitative semi-structured interviews	12 interviews	Five themes identified around the PL was: to document the patient's problems, to make sense of the patient's problems, to make decisions about the care plan, to know who is involved in the patient's care, and to communicate with others. Physicians’ suggested criteria for a PL varied across services with respect to goals, characteristics, and patient-related information. Physicians involved in pediatric trauma care described the electronic PL as ideally more than a list of a patient's medical diagnoses and injuries. The information elements mentioned are typically found in other parts of the patient's electronic record besides the PL, such as past medical history and labs.
Idres, 2025 [48]	Sudan	IM	Retrospective	22 clinical problems (1st cycle), 23 clinical problems (2nd cycle)	Introducing a structured data template improved PL documentation by 16%.
Jackson, 2023 [49]	USA	ED	Retrospective EHR-data set	202 patient records	Gout alert system only had modest predictive value.
Jiggins, 2016 [50]	USA	FP group	EHR-data set	100 clinical summaries	Meaningful Use clinical summaries audit found high incomplete PLs. Multiple barriers to patient engagement were found including health illiteracy and lack of computer use familiarity among the elderly.
Joseph, 2016 [51]	USA	ED	Retrospective EHR-data set	603 patients	Results showed the feasibility of using a simple automated algorithm to derive a PL from the medication list. The algorithm demonstrated greater sensitivity than attending physicians and the independent EHR PL for all conditions. It was less specific for conditions with more heterogeneous management like hypertension and CHF.
Kagan, 2017 [52]	USA	Nursing	Commentary	N/A	The PL and the biomedical model in which it originates lack a framework through which to understand the person and create a complementary inventory of advantages. Every person, no matter how frail and ill, possesses strengths and resources that may offset particular disadvantages or be leveraged to mitigate specific problems. Framing knowledge of the person within a model of care situates an inventory of advantages within the plan of care.
Kieft, 2017 [53]	Netherlands	Nursing	Explorative qualitative focus group	67 participants	119 current and potential patients’ problems were included and defined based on SNOMED CT to assist interoperability within and between EHRs. Because a terminology can contain an enormous number of concepts, subsets are developed to ensure appropriate use in daily practice.
King, 2022 [54]	USA	ED	Retrospective EHR-based data	200 patients	Rule-based algorithm can correctly measure multimorbidity in the ED with moderate-to-good accuracy.
Klappe, 2020 [55]	Netherlands	MC	Qualitative semi-structured interviews	24 interviews	Large variability in attitudes towards PL use. Barriers included uncertainty about the responsibility for PL maintenance and little perceived benefits. Facilitators included the (re)design of policies, improved (peer-to-peer) training to increase motivation, and positive peer feedback and monitoring. Motivation is best increased through sharing benefits relevant in the care process, such as providing overview, timely generation of discharge or referral letters, and reuse of data. Furthermore, content of the underlying terminology should be improved, and the PL should be better presented in the EHR system. To let physicians, accept and use the PL, policies and guidelines should be redesigned and prioritized by supervising staff. Additionally, peer-to-peer training on the benefits of using the PL is needed.
Klappe, 2021 [56]	Netherlands	MC	Retrospective EHR-data set	288 935 diagnoses	A rule-based algorithm in identifying contextual properties in Dutch modified diagnosis descriptions. UnLaTem could be extended with more trigger terms, new rules and the recognition of term order to increase the performance even further. Implementing this into hospital systems can improve the precision of data retrieval and extraction from diagnostic description, which be used for data reuse purposes like CDS.
Klappe, 2023 [111]	Taiwan	IM	RCT	160 participants	Correctly structured PLs lead to better and faster clinical decision-making.
Klein, 2025 [112]	USA	PCP	Retrospective EHR study	41 392 patients	A computable phenotyping approach using EHR PL and related structured data identified hear failure cohorts with differing sensitivity/specificity tradeoffs; findings highlighting limitations of relying on PL alone for case identification.
Krauss, 2016 [57]	USA	PCP	Qualitative	32 PCPs	Subjects’ PLs were highly variable from the sample cases. Physicians most frequently ranked PL items based on their acuity and immediate threat to health. The PL is a physician's mental model of a patient's health status. These mental models were found to vary significantly between physicians, raising questions about whether PLs created by individual physicians can serve their intended purpose to improve care coordination.
Kreuzthaler, 2019 [4]	Austria	N/A	Retrospective EHR-data	6 million coded PL entries	Results showed that traceable semantics were captured on a syntactic level above single characters, addressing the idiosyncratic nature of clinical language. The results shows that meaningful lexical semantics could be captured at this representation level using an exploratory evaluation.
Kreuzthaler, 2024 [58]	Austria	N/A	Retrospective EHR-data	20 525 777	Routine problem list descriptions can be reused for automated ICD-10 code assignment using bi-encoder–based language models; however, performance is constrained by the brevity, inconsistency, and contextual ambiguity of problem list entries. Short descriptions, abbreviations, and missing clinical detail limit coding accuracy and highlight underlying issues with problem list accuracy, granularity, and standardization. Findings underscore the need for improved problem list structure and governance to support downstream secondary uses such as clinical decision support, analytics, and AI-driven automation.
Li, 2018 [59]	USA	ICU	Interrupted time series	3650 pre- and 4344 postimplementations	The PL is an underutilized component of EHRs that can be a source of clinician-structured data representing a patient's condition in real-time. Problem based charting (PBC) tools can integrate PL management into physician workflow.
Li, 2020 [60]	USA	IM/OP	QI	242 330 free-text entries	QI initiative identified and remediated free-text allergens in the EHR as a health system-wide patient safety initiative to improve documentation and ensure consistent CDS, including allergy alerting. Free-text allergen entry is variable, and that a single term can be free texted with hundreds of different variations and misspellings by different providers.
Liang, 2017 [61]	USA	MC	Retrospective EHR-data	399 random adults selected	Using NLP and ML through a ground truth generation process uncovered individual annotator errors accounting for inter-annotator disagreements, and ground truth errors in the initial adjudicated PL. The ground truth errors discovered during vetting consisted of missed problems, missed codes, and erroneously-identified problems.
Liang, 2021 [62]	USA	MC	Retrospective EHR-data	1500 longitudinal records	NLP can further support problem list generation but accuracy is still a big concern.
Liao, 2020 [63]	USA	Peds	QI	N/A	Development and implementing an EHR tool to enhance functionality improved communication and congruity of PL. However, us of the PL was variable among different departments. Additionally, there was a lack of user accountability and comfort with manipulating the PL. It was important to generate cultural and workflow changes by increasing provider engagement of the tool.
Mangin, 2020 [64]	Canada	PCP	Prospective pre- and poststudy	Baseline 17 496 ICD 9 codes in registry over 11-years; postimplementation at 6-months 9278 codes added	Implementation of clinician co-designed, workflow-embedded tools can improve problem coding and quality in EMRs, leading to substantial effectiveness in routine clinical care.
Marquez Fosser, 2017 [65]	Brazil	IM	Pre- and postdeployment	39 239 hospitalizations	The development of a comorbidities subset can lead to more meaningful/actionable problems being added to the EMR and more frequent PL reconciliation.
Martin, 2020 [66]	UK	GP	Qualitative MSc survey and interview	60 online surveys and 17 interviews	GPs feel they do not have the time nor training for keeping a shared approach to a current PL. More action is needed to reduce PL variability and improve the quality of shared information.
Martin, 2021 [67]	UK	GP	Commentary	N/A	Clinicians should have a fundamental shift in the central definition of a PL from “problems” to “health issues”. Secondly, instead of using terms “active” and “inactive” for progression of disease a better term is “present” or “current” and “past” or “previous”.
Mattar, 2017 [68]	USA	FM clinic	Retrospective EHR-data	3868 patients	Obesity is under documented in the primary care setting, those with higher BMO scores and morbidity obese only get documented.
McEvoy, 2018 [69]	USA	MC	Intervention development	4324 charts	Using CDS with population health documentation (PHD) improved the documentation of splenectomies.
McNeely, 2025 [70]	USA	IM	Commentary	N/A	Substance use disorder is not being clearly documented in the PL and prevents detection by PCPs.
Meredith, 2020 [71]	UK	N/A	Commentary	N/A	PL records represent a diverse range of entries, but consideration should be given to entries from various sources to reconcile the list.
Meredith, 2021 [72]	UK	N/A	Commentary	N/A	Digital EHR-based systems derived from a openEHR can provide different views to a problem but a core master list should be utilized, alongside ownership by the most responsible physician to ensure accuracy and completeness.
Meyerhoefer, 2017 [73]	USA	OB/GYN	Mixed methods	Triage survey sample 1203 patients, survey sample 377	Source records are important for reliable, complete, consistent, and easily retrievable points of care information. Greater care should be undertaken in the use of automation techniques prior to implementation to ensure accuracy and reliability of the information provided, especially from multiple interfaces.
Molina, 2025 [74]	USA	ED	Cross-sectional	17 103 encounters	Structured SDoH data enable patient's PL to be edited and updated.
Nasir, 2018 [75]	USA	Peds PCP	Retrospective EHR data review	3998 patients	Chronic problems in the pediatric PC setting is highly prevalent and to ensure optimal quality of patient care, PCPs need to take into account the complexity of the profile of patients visiting such academic clinics.
Nelson, 2018 [76]	USA	MC	Retrospective EHR data review	62 191 patients	Most patients did not have a problem listed when matched to a specific medication order, suggesting PLs were incomplete. Using CDS tools may improve rates of completion.
Nugent, 2025 [77]	USA	Peds	Retrospective EHR data	50 920 patients	20% of recognized hypertension cases were recorded only in free-text notes, indicating underutilization of problem lists.
Pahl, 2016 [78]	USA	Peds	Retrospective EHR-data analysis	102 patients	Asthma was infrequently documented in the PL. There were also significant gaps in terms of people treated for acute chest syndrome (ACS) with the episodes not being documented despite the association between ACS and asthma.
Paridaens, 2026 [116]	Belgium	PCP	Delphi	12 GPs	40 extractable Is can be used to map the completeness of PLs in EMRs by setting up an audit and feedback system, and to develop specific (computer-based) training for GPs.
Patel, 2016 [79]	USA	MC	Retrospective EHR-data analysis	1070 patients	Calcium pyrophosphate dihydrate (CPP) crystal deposition in the articular cartilage can often be seen radiographically as chondrocalcinosis (CC). Patients with CC are often at risk for developing acute attacks of crystal-induced arthritis and/or exaggerated forms of osteoarthritis. It is infrequently documented in the PL and physicians should review available CT scans to include or exclude in their PL.
Porter, 2020 [80]	USA	Peds Hospital	Commentary	N/A	Using a shared decision-making model has significant ethical implications around control of the content. Some of the common issues include: 1) factual errors, 2) outdated problems that have since resolved, 3) patient preference to remove actual problems from the PL, and 4) disagreement between patient and provider over whether a PL item accurately reflects the patient's problem.
Poulos, 2021 [81]	UK	IM	QI	516 patients	Diagnoses and other clinical information stored in a structured way in EHR is extremely useful for supporting clinical decisions, improving patient care and enabling better research but it was found that majority of important diagnoses were not recorded in the structured PL nor mentioned in the free text notes.
Prater, 2019 [82]	USA	Hospice	Retrospective cohort	1185 patients	Dedicated ACP documentation in the PL is associated with fewer admissions in the last 30 days of life for patients with advanced cancer referred to hospice.
Prazeres, 2025 [83]	Portugal	PCP	Retrospective EHR data	10 366 861 patients	Problem lists help monitor chronic disease trends but were dependent on clinical documentation practices.
Rajbhandari, 2018 [84]	USA	Peds	QI	N/A	Setting interventions like an awareness campaign, educational session, reminder pages, feedback loops, EHR upgrades and leadership/clinical support improved the use of the PL in a pediatric hospital. Sustainability was maintained with educational interventions and technology leveraging.
Ridgway, 2021 [85]	USA	OP	Retrospective EHR cohort	778 HIV patients	NLP used in the clinical notes identified high rates of mental illness not documented in structured EMR fields.
Rodriguez, 2017 [86]	Argentina	IM	Descriptive, observational	26 410 queries	Infobuttons can an important resource in improving patient care and decision-making for clinicians when creating a PL
Sandhu, 2024 [87]	Canada	PCP	Retrospective	603 437 hospital records	Level of agreement between solution mapped EHR and hospital DAD for ICD-10 CA data (PL) was low, indicating significant differences between terminology mappings and the coding process.
Sanford, 2021 [88]	USA	OB/Gyn	Descriptive methodology	536 unique documents	216 distinct problems that might be encountered during a routine pregnancy were established based on ACOG guidelines. These problems would serve as triggers within the EHR that can be made available to the clinician at the point of care.
Satti, 2021 [89]	USA	Peds	QI	885 patients	Using a multidisciplinary team to develop a specific intervention that included 1) educational sessions; 2) provision of practice support tools; 3) motivational interview training; 4) engagement of providers and staff for creating a culture of change; and 5) creating a Best Practice Advisory (BPA) in the EMR obesity documentation improved
Sauer, 2024 [90]	USA	IM	Retrospective EHR data	700 patients	Documenting depression or anxiety in COVID patients can lead to better identification of mental health issues.
Senior, 2024 [91]	USA	FM	Retrospective EHR data	50 patients	SNOMED CT concept groupers can accurately reflect clinical systems and used for PL organization but challenges still exist in mapping ICD-10 codes to the same SNOMED CT concept.
Simon, 2025a [117]	USA	PCP	Retrospective EHR data	362 436 patients	PL and medication list can be long with unnecessary items. Attestation that the list has been reviewed does not equate to shorter and less duplicative lists. These findings may indicate that the clinician attestation process during clinical encounters is often done whether or not a problem and medication list has, in fact, been updated. Attestation may be a “check-the-box” activity imposing cognitive burden on clinicians to meet a metric while missing the goal of a focused and uncluttered list.
Simon, 2025b [118]	USA	PCP	Retrospective EHR data	892 329 patients	Diagnoses remain on PL far beyond their expected clinical duration. Minimal evidence of PL management.
Singer, 2016 [9]	Canada	PCP	Retrospective EHR data analysis	18 primary clinics	High variability and low quality of PLs (health condition records) related to 7 common chronic diseases in EMRs exist. There are systematic physician—and clinic-level factors associated with low data quality completeness. Improvements are needed in EMR data quality within the primary care setting.
Singer, 2017 [8]	Canada	PCP	Retrospective EHR data analysis	119 practices in 18 primary clinics	Overall PL completeness was low but highest for diabetes and lowest for insomnia. Fee-for-service clinics generally had lower PL completeness than salaried clinics did for all prescription medications examined. Panel size did not affect PL completeness rates. The low EMR problem list completeness suggests that this field is not reliable for use in QI initiatives or research until higher reliability has been demonstrated.
Sinha, 2017 [6]	USA	MC	Retrospective EHR cohort review	5368 patients	There was a trend for an interaction between follow-up within 14 days and discharge type controlling for age, gender, race/ethnicity, insurance, and number of diagnoses on PL. This also highlights the need for better data collection around SDoH variables centering around the PL.
Smith, 2021 [92]	USA	NICU	Evidence-based methodological framework	N/A	PLs should be clear, concise and organized by order of systems.
Smits, 2016 [93]	Netherlands	Primary HC	Retrospective EHR-data analysis	5712 patients	Frequent attenders have more transient and chronic episodes of care with longer duration linked to PL, likely from low threshold to consult their GPs, with high level of anxiety and low mastery.
Sockolow, 2019 [95]	USA	HHC	Observational field-based	12 admissions	A varying number of the 17 unique problems found across the documents were distributed by document type. Patients were referred to home health care with more clinical problems than were documented in the output documents. The decrease in number and mismatch of problems is an issue which could be addressed with EHR redesign and interoperability capability.
Sockolow, 2021 [94]	USA	HHC	Observational field-based	36 patients	Specific patient problem data changes across the admission process phases resulting in incongruences.
Stein, 2019 [96]	USA	MC	Retrospective EHR-data analysis	122 339 patients	Algorithm was efficient at identifying the presence or absence of XFS compared to conventional approach of assessing only billing codes.
Sutton, 2019 [97]	USA	IM	Commentary	N/A	EHR should consolidate data by 1) juxtaposing to each problem relevant medications and results; 2) annotated information relevant under Assessment and Plan; 3) flow sheet; and 4) reducing physician navigation in the EHR by placing abbreviated versions of interdisciplinary progress notes. There should also be physician communication for each problem at points of transition of care including a problem-oriented discharge summary.
Tan, 2024 [98]	USA	Academic MC	EHR-based encounter data	344 unique patients with dementia diagnosis	It is feasible to implement an EHR algorithm for prospective dementia identification with a high positive predictive value.
Teeple, 2023 [99]	USA	Academic hospital ED	Retrospective EHR data	37 196 unique patients	PL missingness around patient race impacts predictive ability of ML model for ED triaging.
Tomita, 2019 [100]	Japan	Geriatric	Qualitative exploratory factor analyses	204 geriatricians	Most geriatricians use PL for interdisciplinary data sharing. Reasons for incomplete PLs were lack of time and lack of standardization of terminologies regarding observed diseases. The listings of “geriatric syndrome” and “assistance needs in medication management” are crucial for improving PL comprehensiveness among the elderly.
Vera Ramos, 2019 [101]	Austria	Inpatient	Pilot study	5 patients	ICD-10 codes PL entries can be used to train German clinical language with certain degree of quality.
Vivthcarenko, 2021 [102]	USA	PICU	Retrospective mixed methods	96 patients	Physicians do not regularly document any diagnostic uncertainties nor utilize lists effectively. Require further training on how poor documentation can impact downstream patient care and outcomes.
Voss, 2022 [103]	USA	CHC	EHR encounter data	1 180 290 patients	Varying levels of concordance was found between encounter diagnosis and problem list diagnosis for chronic condition status. Problem lists routinely provided higher levels of capture than encounter diagnoses alone.
Wang, 2019 [104]	USA	OP	EHR data set from PCP clinical notes	3.3 million notes with 4701 problems	SDOH data can be a useful guide in patient care and communication between different clinical teams. There is substantial variation in data completion rates and suggests the need for more institutional-level support like EHR-user education and targeted workflow integration to better integrate SDOH data.
Wang, 2020 [105]	USA	IM/OP	Retrospective EHR data analysis	327 695 patients	There was a variable relationship between demographic factors and rates of completeness and duplication. Rates of completeness were positively correlated with disease severity for most diseases. Rates of duplication were consistently positively correlated with disease severity. Incompleteness and duplications are both important issues in problem lists.
Wang, 2021 [106]	USA	IM	EHR data	27 127 patients	We observed substantial variation in the use of different SDOH EHR data types. Notably, social history was rarely used. There is a need for more focused EHR-user education and workflow integration.
Wardell, 2025 [107]	USA	Pediatrics	Retrospective	9621 patients	Implementation of CDSS improved documenting BMI in PL.
Weiskopf, 2019 [108]	USA	IM	EHR data warehouse, chart review and survey-based	275 participants	Varying degrees of agreement were found between the three data sources, with diabetes having the highest level of agreement and stroke the lowest. Sources of disagreement were grouped into research error, healthcare system factors, documentation factors, diagnosis factors, and patient factors.
Wright, 2023 [12]	USA	IM	EHR-data	4 large healthcare systems	An EHR-embedded CDS intervention was effective at improving problem list completeness.
Xu, 2018 [109]	USA	Not given	Commentary	N/A	AI can transform clinical medicine by allowing physicians to sort through large volumes of data in an organized and efficient manner. However, AI interoperability standards and function require continuous refinement.
Zahar, 2018 [110]	USA	IM	Medical education research registry	129 residents	Only 29% listed depression/dysthymia as a primary diagnosis. Only 48% documented depression/dysthymia on the PL 53% connected the presenting symptom (fatigue) with depression.

ACOG, American College of Obstetricians and Gynecologists; ACP, advanced care planning; ACS, acute chest syndrome; AI, artificial intelligence; BMI, body mass index; BPA, best practice award; CC, chondrocalcinosis; CDS, clinical decision support; CHC, community health center; CHF, congestive heart failure; CPP, calcium pyrophosphate dihydrate; CT, computed tomography; DAD, Discharge Abstract Database; dx, diagnosis; ED, emergency department; EHR, electronic health record; EMR, electronic medical record; FMC, family medicine clinic; GP, general practitioner; ICD, International Classification of Diseases; IM, internal medicine; ML-machine language; MSc, Master of Science; N/A, not available; NLP, natural language processing; PBC, problem based charting; PCP, primary care physician; PHD, population health documentation; PL, problem list; PLM, problem list manager; POMR, problem-oriented medical record; QI, quality improvement; RCT, randomized control trial; SDOH, social determinants of health; UnLaTem, Uncertainty, Laterality, Temporality; UK, United Kingdom; USA, United States of America; XFS, exfoliation syndrome.

With this corpus, we wanted to answer what PL characteristics facilitate their use in primary care or primary care-relevant settings.

### Hodge and Narus seven themes updated

#### Benefits of using a problem list

Across the included studies, the PL was consistently described as a core clinical infrastructure supporting safety, coordination, and longitudinal care in primary care settings. Over 60% of studies identified patient safety and care coordination as a central benefit of PL use (*n* = 66; 64%). Benefits most frequently described included improved care coordination [23], safer prescribing via CDS [13, 60, 115], and better cognitive organization of patient information [23, 25, 117, 118]. In primary care, PL were consistently framed as a central orienting structure for longitudinal care, particularly for multimorbidity and preventive management [23, 40, 47, 59, 100]. Beyond direct care delivery, the PL supported quality improvement and research activities in primary care. Accurate PLs enabled practice-level audits, chronic disease surveillance, and evaluation of care processes, although several studies cautioned that these secondary uses depend heavily on data completeness and governance [9, 46, 83]. Importantly, newer primary care-focused studies emphasized that PL-derived analytics are only reliable when documentation responsibilities and workflows are clearly defined [116].

#### The unused problem list

More than half of the studies (*n* = 59; 57%) reported PLs being incomplete, outdated, duplicated, or bypassed in favor of free text. Primary care-specific studies highlighted under-documentation of obesity, mental health conditions, substance use, and social determinants of health, directly limiting CDS, population health, and equity-oriented care [8, 9, 35, 68, 70, 77].

#### Aspects critical to success

Key success factors were addressed in close to 70% (*n* = 71; 69%) including CDS alignment, clinician training, leadership support, and terminology governance. In primary care, low-friction integration into routine visits and minimizing cognitive burden were repeatedly emphasized [11, 13, 55, 56, 64, 107].

#### Policy

Studies (*n* = 41; 40%) called for clearer institutional policies on PL ownership, reconciliation expectations, and downstream reuse (e.g., billing, CDS, or quality improvement). Primary care studies emphasized ambiguity around responsibility when multiple clinicians contributed over time [30, 32, 115].

#### What belongs on the problem list

There was substantial disagreement regarding scope in multiple studies (*n* = 54; 52%). While chronic conditions were universally endorsed [66, 67], primary care studies highlighted gaps in documenting mental health, obesity, substance use, geriatric syndromes, and social determinants of health [23, 25, 38, 46, 50, 57, 66, 92, 97, 100, 104].

#### Who should maintain the problem list

Responsibility was variably assigned to PCPs, care teams, or “most responsible providers” (*n* = 46; 45%). Primary care settings reported tension due to shared care, consultant input, and time constraints [23, 25, 39, 46, 59, 81]. Team-based approaches and clear delineation of roles are essential for sustainable PL maintenance in busy primary care settings.

#### When should the problem list be updated

Evidence consistently indicates that the PL should be actively maintained at every clinical encounter and in real-time as new information becomes available (*n* = 38; 37%). In primary care and longitudinal settings, the PL is often the first-place clinicians review when preparing for a visit and should be updated before, during, and after encounters to reflect evolving diagnoses and care priorities [23, 46, 81, 92]. Across care transitions, studies emphasize structured review of the PL at key points in the patient journey, particularly at admission and discharge, to support continuity and safe handoffs [81]. In acute and specialty contexts, PL entries are frequently updated as soon as diagnostic clarity is achieved, such as replacing presenting symptoms with definitive diagnoses [46], or within hours of admission when clinical data first become available [38]. Team-based workflows further support routine PL review during shift changes and sign-out to ensure shared situational awareness [92]. Collectively, these findings support a primary care-relevant model in which the PL functions as a living clinical artifact, requiring continuous updating rather than episodic reconciliation.

### Emerging technologies

#### Technologies supporting problem list generation

Technologies supporting PL generation were identified in a subset of studies (*n* = 24; 23%). These studies predominantly used NLP or ML to infer clinical problems from unstructured notes, billing data, or procedure codes and to suggest additions to the PL [36, 49, 61, 94, 95, 107]. Generation-focused interventions most commonly targeted accuracy [94, 95, 116], while fewer studies explicitly demonstrated improvements in completeness [12]. In primary care contexts, automated generation was most often framed as a support mechanism for identifying under-documented chronic conditions rather than as a replacement for clinical judgement [36, 41, 49].

#### Technologies supporting problem list curation

PL curation—defined as clinician review, updating, addition, or removal of problems—was the most frequently addressed sub-component, reported (*n* = 57; 55%). Curation strategies included CDS alerts, workflow prompts, structured review tools, and quality improvement initiatives integrating EHR functionality with training and feedback [12, 36, 44, 49, 54, 61, 87, 107, 118]. Curation-focused studies most frequently addressed completeness [54], particularly for chronic disease documentation relevant to primary care. Improvements in accuracy were reported in 31 studies (30%), although several audits noted that documentation behaviors (e.g., marking the PL as “reviewed”) did not consistently result in correction of outdated or inaccurate entries [61]. These findings highlight the limits of technology-enabled curation without clear ownership and accountability within primary care workflows.

#### Technologies supporting problem list reconciliation

Technologies supporting PL reconciliation were frequently identified (*n* = 38 studies; 37%). Reconciliation approaches focused on aligning PL content across encounters, care transitions, and data sources, including comparisons between PLs, registries, billing data, and patient self-report [6, 8, 48, 59, 70, 74, 77, 112, 117]. Reconciliation-focused studies most frequently targeted accuracy (*n* = 33; 32%) and completeness (*n* = 29; 28%) [25, 41]. From a primary care perspective, these studies consistently demonstrated persistent discrepancies following hospital discharge or specialty care, underscoring the challenges of maintaining a coherent longitudinal PL across fragmented systems. Studies also highlighted systematic bias and missingness affecting socially vulnerable populations, with implications for PL-driven decision support [101].

#### Technologies supporting problem list organization

PL organization strategies were identified in multiple studies (*n* = 41; 40%). These interventions included terminology-based grouping (e.g. SNOMED CT hierarchies), problem-oriented documentation models, contextual PL templates, and user interface redesigns intended to improve navigation and cognitive efficiency [43, 46, 47, 54, 56]. These benefits were particularly salient in primary care settings managing multimorbidity, where organized and context-aware PLs supported clinical reasoning more effectively than longer or more exhaustive lists.

## Discussion

This rapid scoping review updated Hodge and Narus’ foundational work on electronic PLs to characterize their role, performance, and evolution within primary care. Across 103 included studies, the findings demonstrate that while PLs remain a foundational element of problem-oriented care, persistent challenges related to accuracy, completeness, and usability continue to limit their clinical value. At the same time, the expanding reuse of PL data for CDS, quality measurement, and emerging AI-enabled applications has intensified the consequences of poor PL data quality, particularly in primary care settings where longitudinal care, multimorbidity, and team-based workflows are the norm.

The findings of this review reinforce the view of the PL as a sociotechnical artifact [10] rather than a purely technical data structure. In primary care, PLs function as shared cognitive tools that support continuity of care across encounters, providers, and care settings [6, 8, 48, 59, 70, 74, 77, 112, 117]. However, variability in documentation practices, ambiguity regarding ownership, and misalignment between PL design and clinical workflows remain pervasive [23, 25, 39, 46, 81]. These challenges are particularly salient in primary care, where clinicians must manage evolving diagnostic uncertainty, preventive care, and chronic disease management within constrained visit times. The literature suggests that PL effectiveness is shaped not only by interface design, but also by local norms, training, and organizational expectations surrounding documentation [24–29, 46, 62, 64, 76, 100, 102, 104, 105].

Despite widespread recognition of the PL's importance, many studies report incomplete, outdated, or inconsistently maintained PL [9, 46, 83]. Conditions commonly encountered in primary care—including chronic diseases, mental health conditions, obesity, substance use disorders, and social determinants of health—were frequently under documented or inconsistently represented [23, 25, 38, 46, 50, 57, 66, 92, 97, 100, 104]. Several studies highlighted that formal indicators, such as marking the PL as “reviewed,” did not reliably correspond to meaningful updates, raising concerns about the validity of PL-related quality measures. These findings point to a disconnect between documentation requirements and clinically meaningful PL maintenance, underscoring the need for approaches that prioritize clinical reasoning and continuity of care over administrative compliance.

Emerging technologies—including NLP, ML, and AI-enabled tools—were increasingly described as mechanisms to support PL generation, curation, reconciliation, and organization. While automated approaches showed promise in identifying potentially missing problems, their effectiveness was constrained by limitations related to diagnostic uncertainty, temporality, contextual nuance, and variability in source documentation [25, 41, 54, 94, 95, 116]. These challenges are particularly pronounced in primary care, where problem definitions evolve over time and diagnoses may remain provisional.

In contrast, technologies designed to augment clinician-led workflows—such as tools that prompt review, surface inconsistencies, or support reconciliation across care transitions—were more consistently associated with improvements in PL accuracy, completeness, and usability [25, 41, 54, 94, 95, 116]. The literature suggests that AI-enabled PL tools are most effective when they support, rather than replace, clinical judgment [46]. Without clear governance structures, standardized terminology, and integration into routine workflows, automation risks perpetuating existing documentation gaps or introducing new sources of error. PCPs therefore play a critical role in guiding the implementation of emerging technologies to ensure that PL innovations remain patient-centered and clinically meaningful [3, 5, 10].

### Integration with primary care

Beyond system design, this review highlights the importance of targeted staff training to embed PL use into routine primary care workflows. Several studies suggest that PL inaccuracies persist not solely due to technical limitations, but because clinicians and staff lack clear guidance on when, how, and by whom PL updates should occur during everyday care [23, 46, 81, 92]. Training that situates PL maintenance within routine clinical activities: such as medication reconciliation, chronic disease follow-up, preventive care visits, care transitions, and team-based case reviews; may help normalize PL updating as an integral component of care rather than an episodic documentation task [119]. Importantly, effective training extends beyond physicians to include nurses, pharmacists, and administrative staff who interact with PLs at different points in the care process and multidisciplinary approaches that clarify shared responsibility for PL identification, verification, and reconciliation have been associated with improved usability and reduced cognitive burden on individual clinicians [3, 5, 10].

Taken together, the findings of this review suggest several actionable implications for primary care. First, PL maintenance should be conceptualized as a shared, team-based responsibility, rather than an implicit task assigned solely to physicians. Clear role delineation across multidisciplinary teams may enhance PL accuracy while reducing individual documentation burden [3, 5, 10]. Second, EHR design improvements should prioritize usability features that support problem-oriented views, reduce duplication, and surface relevant contextual information for patients with complex needs [3, 5, 10]. Third, primary care clinicians should play an active role in shaping the deployment of AI-enabled PL tools, ensuring that automation is used to support clinical judgment, equity, and continuity of care rather than as a substitute for clinician engagement [3, 5, 10].

### Future research and direction

Future research should focus on evaluating governance models that align PL documentation responsibilities, workflows, and downstream data uses in primary care. Greater attention to interdisciplinary collaboration, patient engagement, and equity-oriented documentation practices is needed. As AI-enabled applications continue to evolve, rigorous assessment of how PL data quality influences algorithmic performance, bias, and clinical impact will be essential. Updating foundational PL frameworks to explicitly represent emerging technologies and sociotechnical accountability represents an important direction for advancing problem-oriented care in primary care settings.

### Strengths and limitations

This rapid scoping review [16–18] synthesizes a large and methodologically diverse body of literature to characterize the role and performance of electronic PLs in primary care, drawing on both foundational conceptual work and recent empirical studies. An *a priori* conceptual framework was used to guide data extraction and synthesis, enabling consistent comparison across heterogeneous study designs and supporting transparent mapping of findings related to PL accuracy, completeness, and usability. Primary care relevance was operationalized broadly to include settings where PL use is conceptually or operationally transferable, which strengthened ecological validity but may limit specificity to traditional general practice contexts.

The review relied on a single bibliographic database, which may limit comprehensiveness despite alignment with guidance for rapid evidence synthesis in evolving fields. In addition, reliance on an *a priori* framework may have constrained interpretive independence and reproducibility, as alternative frameworks or inductive analytic approaches could yield different thematic emphases. Although the use of a modified JBI checklist enhanced transparency across diverse study designs, reliance on an adapted appraisal framework may limit reproducibility, as alternative appraisal tools or inductive approaches could yield different methodological interpretations. Finally, variability in study designs, clinical settings, and outcome measures limited direct comparison of interventions and precluded conclusions regarding effectiveness.

## Conclusion

High-quality electronic PLs remain foundational to effective primary care, yet their value depends on governance, workflow integration, and shared accountability rather than technology alone. Updating the Hodge and Narus framework to explicitly incorporate emerging AI-enabled tools underscores that meaningful improvement in PL accuracy, completeness, and usability must be led by primary care teams and embedded within everyday clinical practice.

## Supplementary Material

cmag036_Supplementary_Data

## Data Availability

The data underlying this article will be shared on reasonable request to the corresponding author.

## References

[1] Weed LL . What physicians worry about: how to organize care of multiple-problem patients. Mod Hosp 1968;110:90–4. PMID: 5705976. 5705976

[2] Weed LL . Medical records that guide and teach. N Engl J Med 1968;278:652–7. 10.1056/NEJM196803212781204 5637250

[3] Simons SM, Cillessen FHJM, Hazelzet JA. Determinants of a successful problem list to support the implementation of the problem-oriented medical record according to recent literature. BMC Med Inform Decis Mak 2016;16:102. 10.1186/s12911-016-0341-0 27485127 PMC4970280

[4] *Kreuzthaler M, Pfeifer B, Vera Ramos JA et al EHR problem list clustering for improved topic-space navigation. BMC Med Inform Decis Mak 2019;19:72. 10.1186/s12911-019-0789-9 30943968 PMC6448176

[5] Shah AD, Quinn NJ, Chaudhry A et al Recording problems and diagnoses in clinical care: developing guidance for healthcare professionals and system designers. BMJ Health Care Inform 2019;26:e100106. 10.1136/bmjhci-2019-100106 PMC7062352 31874855

[6] *Sinha S, Seirup J, Carmel A. Early primary care follow-up after ED and hospital discharge—does it affect readmissions? Hosp Pract (1995) 2017;45:51–7. 10.1080/21548331.2017.1283935 28095063

[7] Azamar-Alonso A, Costa AP, Huebner L-A et al Electronic referral systems in health care: a scoping review. Clinicoecon Outcomes Res 2019;11:325–33. 10.2147/CEOR.S195597 31190925 PMC6511625

[8] *Singer A, Kroeker AL, Yakubovich S et al Data quality in electronic medical records in Manitoba: do problem lists reflect chronic disease as defined by prescriptions? Can Fam Physician 2017;63:382–9. PMID: 28500199. 28500199 PMC5429058

[9] *Singer A, Yakubovich S, Kroeker AL et al Data quality of electronic medical records in Manitoba: do problem lists accurately reflect chronic disease billing diagnoses? J Am Med Inform Assoc 2016;23:1107–12. 10.1093/jamia/ocw013 27107454 PMC11960764

[10] Hodge CM, Narus SP. Electronic problem lists: a thematic analysis of a systematic literature review to identify aspects critical to success. J Am Med Inform Assoc 2018;25:603–13. 10.1093/jamia/ocy011 29547974 PMC7647009

[11] *Altman RL, Lin C-T, Earnest M. Problem-oriented documentation: design and widespread adoption of a novel toolkit in a commercial electronic health record. JAMIA Open 2023;6:ooad005. 10.1093/jamiaopen/ooad005 36751467 PMC9897179

[12] *Wright A, Schreiber R, Bates DW et al A multi-site randomized trial of a clinical decision support intervention to improve problem list completeness. J Am Med Inform Assoc 2023;30:899–906. 10.1093/jamia/ocad020 36806929 PMC10114117

[13] *Grauer A, Kneifati-Hayek J, Reuland B et al Indication alerts to improve problem list documentation. J Am Med Inform Assoc 2022;29:909–17. 10.1093/jamia/ocab285 34957491 PMC9006708

[14] Abbasgholizadeh Rahimi S, Légaré F, Sharma G et al Application of artificial intelligence in community-based primary health care: systematic scoping review and critical appraisal. J Med Internet Res 2021;23:e29839. 10.2196/29839 34477556 PMC8449300

[15] Andrew A . Potential applications and implications of large language models in primary care. Fam Med Community Health 2024;12:e002602. 10.1136/fmch-2023-002602 38290759 PMC10828839

[16] Tricco AC, Antony J, Zarin W et al A scoping review of rapid review methods. BMC Med 2015;13:224. 10.1186/s12916-015-0465-6 26377409 PMC4574114

[17] Campbell F, Sutton A, Pollock D et al Rapid reviews methods series: guidance on rapid scoping, mapping and evidence and gap map (“big picture reviews”). BMJ Evid Based Med 2025;30:268–77. 10.1136/bmjebm-2023-112389 PMC12320599 39904600

[18] Hamel C, Michaud A, Thuku M et al Defining rapid reviews: a systematic scoping review and thematic analysis of definitions and defining characteristics of rapid reviews. J Clin Epidemiol 2021;129:74–85. 10.1016/j.jclinepi.2020.09.041 33038541

[19] Tricco AC, Lillie E, Zarin W et al PRISMA extension for scoping reviews (PRISMA-ScR): checklist and explanation. Ann Intern Med 2018;169:467–73. 10.7326/M18-0850 30178033

[20] Tricco AC, Thomas SM, Antony J et al Strategies to prevent or reduce gender bias in peer review of research grants: a rapid scoping review. PLoS One 2017;12:e0169718. 10.1371/journal.pone.0169718 28061509 PMC5218731

[21] McGowan J, Sampson M, Salzwedel DM et al PRESS peer review of electronic search strategies: 2015 guideline statement. J Clin Epidemiol 2016;75:40–6. 10.1016/j.jclinepi.2016.01.021 27005575

[22] Thomas J, Harden A. Methods for the thematic synthesis of qualitative research in systematic reviews. BMC Med Res Methodol 2008;8:45. 10.1186/1471-2288-8-45 18616818 PMC2478656

[23] *Borbolla D, Taft T, Taber P et al Understanding primary care providers’ information gathering strategies in the care of children and youth with special health care needs. AMIA Annu Symp Proc 2018;2018:272–8. PMID: 30815065. 30815065 PMC6371377

[24] *Bowles KH, Stawnychy MA, O’Connor M et al Context and determinants for implementing a sepsis survivor care transition intervention reported from five health systems and home health agencies. Front Med (Lausanne) 2025;12:1632083. 10.3389/fmed.2025.1632083 41404587 PMC12702754

[25] *Brown AR, McCoy AB, Wright A et al Decluttering the problem list in electronic health records. Am J Health Syst Pharm 2022;79:S8–12. 10.1093/ajhp/zxab381 34597358

[26] *Budde AM, Barrett AL, Benner AC et al Difficult intubation alert is associated with a reduced incidence of difficult intubation. Cureus 2024;16:e72625. 10.7759/cureus.72625 39610643 PMC11604021

[27] *Buttafuoco KA, Mokshagundam S, Henricks A et al Impact of electronic medical record utilization on obesity screening and intervention for obese patients with endometrial cancer. Int J Gynecol Cancer 2024;34:830–9. 10.1136/ijgc-2023-005247 38519088 PMC11187359

[28] *Ceusters W, Blaisure J. Caveats for the use of the active problem list as ground truth for decision support. Stud Health Technol Inform 2018;255:10–4. 10.3233/978-1-61499-921-8-10 30306897

[29] *Chehal PK, Dieci M, Li Z et al The probability of preterm or early term second live births in the southern U.S. state of Georgia, 2011–2020. BMC Pregnancy Childbirth 2025;25:814. 10.1186/s12884-025-07763-1 40770789 PMC12326856

[30] *Chen C-C, Chang C-H, Peng Y-C et al Effect of implementation of a coded problem list entry subsystem. Comput Methods Programs Biomed 2016;134:1–9. 10.1016/j.cmpb.2016.05.012 27480728

[31] *Chen C-Y, Chen Y-L, Scholl J et al Ability of machine-learning based clinical decision support system to reduce alert fatigue, wrong-drug errors, and alert users about look alike, sound alike medication. Comput Methods Programs Biomed 2024;243:107869. 10.1016/j.cmpb.2023.107869 37924770

[32] *Cillessen F, de Vries Robbé P, Bor H et al Factors affecting the manual linking of clinical progress notes to problems in daily clinical practice: a retrospective quantitative analysis and cross sectional survey. Health Inform J 2021;27:14604582211007534. 10.1177/14604582211007534 33840302

[33] *Cillessen F, Hofdijk J. Transition requirements from problem list to an overarching care plan for the support of person-centered care. Stud Health Technol Inform 2020;272:292–5. 10.3233/SHTI200552 32604659

[34] *Cohen GR, Friedman CP, Ryan AM et al Variation in physicians’ electronic health record documentation and potential patient harm from that variation. J Gen Intern Med 2019;34:2355–67. 10.1007/s11606-019-05025-3 31183688 PMC6848521

[35] *Cyr PR, Haskins AE, Holt C et al Weighty problems: predictors of family physicians documenting overweight and obesity. Fam Med 2016;48:217–21. PMID: 26950911. 26950911

[36] *Daskivich TJ, Abedi G, Kaplan SH et al Electronic health record problem lists: accurate enough for risk adjustment? Am J Manag Care 2018;24:e24–9. PMID: 29350512. 29350512

[37] *Devarakonda MV, Mehta N, Tsou C-H et al Automated problem list generation and physicians’ perspective from a pilot study. Int J Med Inform 2017;105:121–9. 10.1016/j.ijmedinf.2017.05.015 28750905

[38] *Doghramji K, Tanielian M, Certa K et al Severity, prevalence, predictors, and rate of identification of insomnia symptoms in a sample of hospitalized psychiatric patients. J Nerv Ment Dis 2018;206:765–9. 10.1097/NMD.0000000000000888 30273272

[39] *Durojaiye AB, McGerorge N, Kristen W et al Characterizing the utilization of the problem list for pediatric trauma care. AMIA Annu Symp Proc 2018;2018:404–12. PMID: 3081508. 30815080 PMC6371241

[40] *Flanagan MR, Foster CC, Schleyer A et al Aligning institutional priorities: engaging house staff in a quality improvement and safety initiative to fulfill clinical learning environment review objectives and electronic medical record meaningful use requirements. Am J Surg 2016;211:390–7. 10.1016/j.amjsurg.2015.09.006 26687964

[41] *Gammal RS, Berenbrok LA, Empey PE et al Documenting pharmacogenomic test results in electronic health records: practical considerations for primary care teams. J Pers Med 2021;11:1296. 10.3390/jpm11121296 34945768 PMC8706275

[42] *Harris DR, Henderson DW, Corbeau A. Improving the utility of tobacco-related problem list entries using natural language processing. AMIA Annu Symp Proc 2020;2020:534–43. PMID: 33936427. 33936427 PMC8075422

[43] *Heintzman J, Kaufmann J, Lucas J et al Asthma care quality, language, and ethnicity in a multi-state network of low-income children. J Am Board Fam Med 2020;33:707–15. 10.3122/jabfm.2020.05.190468 32989065 PMC8682951

[44] *Hier DB, Pearson J. Two algorithms for the reorganisation of the problem list by organ system. BMJ Health Care Inform 2019;26:e100024. 10.1136/bmjhci-2019-100024 PMC7062335 31848142

[45] *Hodge CM, Narus SP, Stoddard G. Developing and validating a model for detecting longitudinal inconsistencies in the electronic problem list. AMIA Annu Symp Proc 2020;2020:563–72. PMID: 33936430. 33936430 PMC8075429

[46] *Horng S, Greenbaum NR, Nathanson LA et al Consensus development of a modern ontology of emergency department presenting problems–the Hierarchical Presenting Problem Ontology (HaPPy). Appl Clin Inform 2019;10:409–20. 10.1055/s-0039-1691842 31189204 PMC6561773

[47] *Hose B-Z, Hoonakker PLT, Wooldridge AR et al Physician perceptions of the electronic problem list in pediatric trauma care. Appl Clin Inform 2019;10:113–22. 10.1055/s-0039-1677737 30759492 PMC6374147

[48] *Idres MOM, Osman OME, Salih HZF et al Evaluating and improving the quality of clinical documentation using the Background, Subjective, Objective, Assessment, and Plan (BSOAP) format: a clinical audit at El-Gadarif Teaching Hospital. Cureus 2025;17:e90580. 10.7759/cureus.90580 40984914 PMC12450290

[49] *Jackson LE, Annapureddy N, Hansen ME et al Development and validation of an emergency department electronic medical record gout flare alert. Arthritis Care Res (Hoboken) 2023;75:1821–9. 10.1002/acr.25061 36408730 PMC10500930

[50] *Jiggins K . A content analysis of the meaningful use clinical summary: do clinical summaries promote patient engagement? Prim Health Care Res Dev 2016;17:238–51. 10.1017/S1463423615000353 26189510

[51] *Joseph JW, Chiu DT, Nathanson LA et al A rules based algorithm to generate problem lists using emergency department medication reconciliation. Int J Med Inform 2016;94:117–22. 10.1016/j.ijmedinf.2016.06.008 27573319

[52] *Kagan SH . Balancing the problem list with an advantage inventory. Geriatr Nurs 2017;38:157–9. 10.1016/j.gerinurse.2017.03.003 28318783

[53] *Kieft RAMM, Vreeke EM, de Groot EM et al The development of a nursing subset of patient problems to support interoperability. BMC Med Inform Decis Mak 2017;17:158. 10.1186/s12911-017-0567-5 29202818 PMC5716238

[54] *King BL, Meyer ML, Chari SV et al Accuracy of the electronic health record's problem list in describing multimorbidity in patients with heart failure in the emergency department. PLoS One 2022;17:e0279033. 10.1371/journal.pone.0279033 36512600 PMC9747000

[55] *Klappe ES, de Keizer NF, Cornet R. Factors influencing problem list use in electronic health records–application of the unified theory of acceptance and use of technology. Appl Clin Inform 2020;11:415–26. 10.1055/s-0040-1712466 32521555 PMC7286722

[56] *Klappe ES, van Putten FJP, de Keizer NF et al Contextual property detection in Dutch diagnosis descriptions for uncertainty, laterality and temporality. BMC Med Inform Decis Mak 2021;21:120. 10.1186/s12911-021-01477-y 33827555 PMC8028823

[57] *Krauss JC, Boonstra PS, Vantsevich AV et al Is the problem list in the eye of the beholder? An exploration of consistency across physicians. J Am Med Inform Assoc 2016;23:859–65. 10.1093/jamia/ocv211 27002075 PMC4997039

[58] *Kreuzthaler M, Pfeifer B, Schulz S. Secondary use of clinical problem list descriptions for bi-encoder based ICD-10 classification. AMIA Annu Symp Proc 2024;2024:620–7. PMID: 40417589. 40417589 PMC12099355

[59] *Li RC, Garg T, Cun T et al Impact of problem-based charting on the utilization and accuracy of the electronic problem list. J Am Med Inform Assoc 2018;25:548–54. 10.1093/jamia/ocy007 29360995 PMC6018915

[60] *Li L, Foer D, Hallisey RK et al Improving allergy documentation: a retrospective electronic health record system-wide patient safety initiative. J Patient Saf 2020;16:e1–7. 10.1097/PTS.0000000000000242 32487880 PMC7704710

[61] *Liang JJ, Tsou C-H, Devarakonda MV. Ground truth creation for complex clinical NLP tasks—an iterative vetting approach and lessons learned. AMIA Jt Summits Transl Sci Proc 2017;2017:203–12. PMID: 28815130. 28815130 PMC5543376

[62] *Liang JJ, Tsou CH, Dandala B et al Reducing physicians’ cognitive load during chart review: a problem-oriented summary of the patient electronic record. AMIA Annu Symp Proc 2021;2021:763–72. PMID: 35308927. 35308927 PMC8861663

[63] *Liao N, Kasick R, Allen K et al Pediatric inpatient problem list review and accuracy improvement. Hosp Pediatr 2020;10:941–8. 10.1542/hpeds.2020-0059 33051244

[64] *Mangin D, Lawson J, Adamczyk K et al Embedding “smart” disease coding within routine electronic medical record workflow: prospective single-arm trial. JMIR Med Inform 2020;8:e16764. 10.2196/16764 32716304 PMC7418012

[65] *Fosser SM, Gaiera A, Otero C et al Automatic loading of problems using a comorbidities subset: one step to organize and maintain the patient's problem list. Stud Health Technol Inform 2017;245:1358. 10.3233/978-1-61499-830-3-1358 29295437

[66] *Martin PM, Sbaffi L. Electronic health record and problem lists in Leeds, United Kingdom: variability of general practitioners’ views. Health Inform J 2020;26:1898–911. 10.1177/1460458219895184 31875417

[67] *Millares Martin P . The problem with the “problem list” name. J Med Syst 2021;45:93. 10.1007/s10916-021-01777-9 34515849

[68] *Mattar A, Carlston D, Sariol G et al The prevalence of obesity documentation in primary care electronic medical records: are we acknowledging the problem? Appl Clin Inform 2017;8:67–79. 10.1055/s-0036-1584227 28119990 PMC5373753

[69] *McEvoy D, Gandhi TK, Turchin A et al Enhancing problem list documentation in electronic health records using two methods: the example of prior splenectomy. BMJ Qual Saf 2018;27:40–7. 10.1136/bmjqs-2017-006839 28754813

[70] *McNeely J, Bradley KA, Liebschutz JM et al Why is substance use missing from my patient's problem list? CTN research to advance screening, prevention, and treatment of substance use in primary care. J Subst Use Addict Treat 2025:209780. 10.1016/j.josat.2025.209780 40782845 PMC12427610

[71] *Meredith J, McNicoll I, Whitehead N et al Defining the contextual problem list. Stud Health Technol Inform 2020;270:567–71. 10.3233/SHTI200224 32570447

[72] *Meredith J, McNicoll I, Whitehead N et al openEHR-based contextual problem list. Stud Health Technol Inform 2021;281:490–1. 10.3233/SHTI210209 34042615

[73] *Meyerhoefer CD, Sherer SA, Deily ME et al A mixed methods study of clinical information availability in obstetric triage and prenatal offices. J Am Med Inform Assoc 2017;24:e87–94. 10.1093/jamia/ocw113 27539200 PMC7651939

[74] *Molina MF, Pimentel SD, Fenton C et al Characterizing emergency clinician engagement with social drivers of health data among patients with opioid use disorder. medRxiv, 10.64898/2025.12.15.25342249, 17 December 2025, preprint: not peer reviewed. PMC13580726 42310717

[75] *Nasir A, Nasir L, Tarrell A et al Complexity in pediatric primary care. Prim Health Care Res Dev 2018;20:e59. 10.1017/S146342361800035X 29785895 PMC8512535

[76] *Nelson SD, Woodroof T, Liu W et al Link between prescriptions and the electronic health record. Am J Health Syst Pharm 2018;75:S29–34. 10.2146/ajhp170455 29802176

[77] *Nugent JT, Cueto V, Tong C et al Accuracy of electronic health record phenotypes to detect recognition of hypertension in pediatric primary care. Acad Pediatr 2025;25:102629. 10.1016/j.acap.2024.102629 39732164 PMC11893226

[78] *Pahl K, Mullen CA. Acute chest syndrome in sickle cell disease: effect of genotype and asthma. Exp Biol Med (Maywood) 2016;241:745–58. 10.1177/1535370216636720 26936083 PMC4950378

[79] *Patel T, Ryan L, Dubois M et al The prevalence of chondrocalcinosis of the symphysis pubis on CT scan and correlation with calcium pyrophosphate dihydrate crystal deposition disease. Clin Rheumatol 2016;35:771–3. 10.1007/s10067-016-3193-1 26861035

[80] *Porter AS, O’Callaghan J, Englund KA et al Problems with the problem list: challenges of transparency in an era of patient curation. J Am Med Inform Assoc 2020;27:981–4. 10.1093/jamia/ocaa040 32346726 PMC7647313

[81] *Poulos J, Zhu L, Shah AD. Data gaps in electronic health record (EHR) systems: an audit of problem list completeness during the COVID-19 pandemic. Int J Med Inform 2021;150:104452. 10.1016/j.ijmedinf.2021.104452 33864979 PMC9759969

[82] *Prater LC, Wickizer T, Bower JK et al The impact of advance care planning on end-of-life care: do the type and timing make a difference for patients with advanced cancer referred to hospice? Am J Hosp Palliat Care 2019;36:1089–95. 10.1177/1049909119848987 31088134

[83] *Prazeres F . Nationwide study on multimorbidity prevalence: 7.64 million primary healthcare users in Portugal with multiple chronic conditions. Public Health 2025;240:18–20. 10.1016/j.puhe.2025.01.005 39848032

[84] *Rajbhandari P, Auron M, Worley S et al Improving documentation of inpatient problem list in electronic health record: a quality improvement project. J Patient Saf 2018;17:e1371–5. 10.1097/PTS.0000000000000490 29672356

[85] *Ridgway JP, Uvin A, Schmitt J et al Natural language processing of clinical notes to identify mental illness and substance use among people living with HIV: retrospective cohort study. JMIR Med Inform 2021;9:e23456. 10.2196/23456 33688848 PMC7991991

[86] Rodriguez JF, Mole ES, Rubin L et al Bringing knowledge to users in one click: infobuttons in the problem list of an EHR. Stud Health Technol Inform 2017;245:1283. 10.3233/978-1-61499-830-3-1283 29295368

[87] *Sandhu N, Onos D, Li B et al How does the mapped ICD data in an EHR system compare to the hospital DAD data in Alberta, Canada? BMC Health Serv Res 2025;25:1523. 10.1186/s12913-025-13716-3 41291697 PMC12648991

[88] *Sanford BH, Labbad G, Hersh AR et al Leveraging American College of Obstetricians and Gynecologists guidelines for point-of-care decision support in obstetrics. Appl Clin Inform 2021;12:800–7. 10.1055/s-0041-1733933 34470056 PMC8410237

[89] *Satti KF, Tanski SE, Jiang Y et al Improving care for childhood obesity: a quality improvement initiative. Pediatr Qual Saf 2021;6:e412. 10.1097/pq9.0000000000000412 34046541 PMC8143745

[90] *Sauer MC, Barlow PB, Comellas AP et al Anxiety and depression symptoms among patients with long COVID: a retrospective cohort study. Eur Arch Psychiatry Clin Neurosci 2024;274:1879–86. 10.1007/s00406-023-01740-5 38231397

[91] *Senior R, Tsai T, Ratliff W et al Evaluation of SNOMED CT grouper accuracy and coverage in organizing the electronic health record problem list by clinical system: observational study. JMIR Med Inform 2024;12:e51274. 10.2196/51274 38836556 PMC11151346

[92] *Smith M, Bosque E. Neonatal care provider tasks in the NICU and delivery room. Adv Neonatal Care 2022;22:215–22. 10.1097/ANC.0000000000000917 34334678

[93] *Smits FT, Brouwer HJ, Schene AH et al Is frequent attendance of longer duration related to less transient episodes of care? A retrospective analysis of transient and chronic episodes of care. BMJ Open 2016;6:e012563. 10.1136/bmjopen-2016-012563 PMC5168647 27965250

[94] *Sockolow PS, Bowles KH, Le NB et al There's a problem with the problem list: incongruence of patient problem information across the home care admission. J Am Med Dir Assoc 2021;22:1009–14. 10.1016/j.jamda.2020.06.032 32736995

[95] *Sockolow PS, Le NB, Yang Y et al Incongruence of patient problem information across three phases of home care admission: there's a problem with the problem list. Stud Health Technol Inform 2019;264:803–7. 10.3233/SHTI190334 31438035

[96] *Stein JD, Rahman M, Andrews C et al Evaluation of an algorithm for identifying ocular conditions in electronic health record data. JAMA Ophthalmol 2019;137:491–7. 10.1001/jamaophthalmol.2018.7051 30789656 PMC6512255

[97] *Sutton JM, Ash SR, Al Makki A et al A daily hospital progress note that increases physician usability of the electronic health record by facilitating a problem-oriented approach to the patient and reducing physician clerical burden. Perm J 2019;23:18-221. 10.7812/TPP/18-221 PMC6636503 31314721

[98] *Tan ZS, Qureshi N, Roberts P et al Alerting providers to hospitalized persons with dementia using the electronic health record. J Am Geriatr Soc 2024;72:822–7. 10.1111/jgs.18673 37937688

[99] *Teeple S, Smith A, Toerper M et al Exploring the impact of missingness on racial disparities in predictive performance of a machine learning model for emergency department triage. JAMIA Open 2023;6:ooad107. 10.1093/jamiaopen/ooad107 38638298 PMC11025382

[100] *Tomita N, Kojima T, Ishiki A et al Could problem lists summarize comprehensive geriatric assessments? A nationwide cross-sectional survey on geriatricians’ attitudes towards problem lists. Geriatr Gerontol Int 2019;19:159–64. 10.1111/ggi.13574 30556272

[101] *Vera Ramos JA, Kreuzthaler M, Schulz S. Supervised ICD code assignment to short clinical problem list entries. Stud Health Technol Inform 2019;258:184–8. 10.3233/978-1-61499-959-1-184 30942742

[102] *Vivtcharenko VY, Ramesh S, Dukes KC et al Diagnosis documentation of critically ill children at admission to a PICU. Pediatr Crit Care Med 2022;23:99–108. 10.1097/PCC.0000000000002812 34534163 PMC8816809

[103] *Voss RW, Schmidt TD, Weiskopf N et al Comparing ascertainment of chronic condition status with problem lists versus encounter diagnoses from electronic health records. J Am Med Inform Assoc 2022;29:770–8. 10.1093/jamia/ocac016 35165743 PMC9006679

[104] *Wang L, Wang Y, Shen F et al Discovering associations between problem list and practice setting. BMC Med Inform Decis Mak 2019;19:69. 10.1186/s12911-019-0779-y 30943957 PMC6448189

[105] *Wang EC-H, Wright A. Characterizing outpatient problem list completeness and duplications in the electronic health record. J Am Med Inform Assoc 2020;27:1190–7. 10.1093/jamia/ocaa125 32620950 PMC7481031

[106] *Wang M, Pantell MS, Gottlieb LM et al Documentation and review of social determinants of health data in the EHR: measures and associated insights. J Am Med Inform Assoc 2021;28:2608–16. 10.1093/jamia/ocab194 34549294 PMC8633631

[107] *Wardell JR, Shaska N, Ahmed Z et al Leveraging clinical decision support system tools for childhood overweight/obesity management. BMC Med Inform Decis Mak 2025;26:19. 10.1186/s12911-025-03293-0 41388408 PMC12822073

[108] *Weiskopf NG, Cohen AM, Hannan J et al Towards augmenting structured EHR data: a comparison of manual chart review and patient self-report. AMIA Annu Symp Proc 2020;2019:903–12. PMID: 32308887. 32308887 PMC7153078

[109] *Xu S, Papier A. Returning to (electronic) health records that guide and teach. Am J Med 2018;131:723–5. 10.1016/j.amjmed.2017.12.048 29427581

[110] *Zabar S, Hanley K, Watsula-Morley A et al Using unannounced standardized patients to explore variation in care for patients with depression. J Grad Med Educ 2018;10:285–91. 10.4300/JGME-D-17-00736.1 29946385 PMC6008039

[111] *Klappe ES, Heijmans J, Groen K et al Correctly structured problem lists lead to better and faster clinical decision-making in electronic health records compared to non-curated problem lists: a single-blinded crossover randomized controlled trial. Int J Med Inform 2023;180:105264. 10.1016/j.ijmedinf.2023.105264 37890203

[112] *Klein S, Mukhopadhyay A, Hamo CE et al Accuracy of electronic health record-based definitions for patients with heart failure. Am J Med 2025;138:1557–1560.e1. 10.1016/j.amjmed.2025.07.010 40684967 PMC12300641

[113] *Campbell A, Bailey SR, Hoffman KA et al Associations between psychiatric disorders and cannabis-related disorders documented in electronic health records. J Psychoactive Drugs 2020;52:228–36. 10.1080/02791072.2020.1747665 32295501 PMC7671705

[114] *Callahan KE, Wilson LA, Pavon JM et al Internal medicine residents’ ambulatory management of core geriatric conditions. J Grad Med Educ 2017;9:338–44. 10.4300/JGME-D-16-00428.1 28638514 PMC5476385

[115] *Capito JE, Dilcher BZ, Jafary ZI. Effect of clinical decision support alerts on anticoagulation management in atrial fibrillation. Appl Clin Inform 2025;16:1892–9. 10.1055/a-2765-6969 41412451 PMC12714456

[116] *Paridaens R, Van den Bulck S, De Jonghe M et al Development of quality indicators for the correct use of electronic medical records in primary care: modified Delphi study. JMIR Med Inform 2026;14:e80057. 10.2196/80057 41553117 PMC12865340

[117] *Simon J, Panzer J, Ekong A et al Problem and medication list review: more than checking a box? Qual Manag Health Care 2025a. 10.1097/QMH.0000000000000530 41307509

[118] *Simon J, Panzer J, Ekong A et al Checking the box: the association between “problem list reviewed” and outdated diagnoses on the list. Appl Clin Inform 2025b;16:1779–86. 10.1055/a-2735-0587 41265889 PMC12634207

[119] Elma A, Jeyabalan V, Wilson E et al Compassionate primary care training and practice in the digital technology sphere: a scoping review protocol. Can Med Educ J 2025;16:54–8. 10.36834/cmej.81831 41584936 PMC12826808

