# Supplementary material for: Current state of electronic problems lists in primary care: a rapid scoping review
Source: Fam Pract. 2026 Jun 11;43(4):cmag036. doi: 10.1093/fampra/cmag036 (PMC13255725; doi:10.1093/fampra/cmag036)
Supplement: cmag036_Supplementary_Data [file cmag036_supplementary_data.zip › Supplemental Files.pdf]

Supplemental Table S1. Full Search Strategy from March 11, 2016 to May 15, 2026.

Search strategy	Results (n)
Medical Records/ or medical records*.mp.	222,819
Medical Records, Problem-Oriented/ or problem oriented*.mp.	3,014
problem list*.mp.	1,132
problem oriented record*.mp.	136
problem oriented chart*.mp.	19
problem summary list*.mp.	5
problem oriented patient record*.mp.	6
problem oriented system*.mp.	79
weed system*.mp.	23
exp Artificial Intelligence/ or Artificial Intelligence.mp. or exp Natural Language Processing/ or (exp Machine Learning/ or Machine Learning.mp.)	439,549
1 or 2 or 3 or 4 or 5 or 6 or 7 or 8 or 9	224,882
10 and 11	4,258

Supplemental Table S2. Modified* JBI Critical Appraisal for Systematic Reviews and Research Synthesis results.

First Author, Year	Q1	Q2	Q3	Q4	Q5	Q6	Q7	Q8	Q9	Q10	Q11
Altman, 2023	Y	Y	Y	Y	Y	Y	Y	Y	N	Y	Y
Borbolla, 2018	Y	Y	Y	Y	U	Y	Y	Y	N	Y	Y
Bowles, 2025	Y	Y	Y	Y	U	Y	Y	Y	N	Y	Y
Brown, 2021	Y	Y	Y	Y	Y	Y	Y	N	Y	Y	Y
Budde, 2024	Y	Y	Y	N	Y	Y	Y	Y	N	Y	Y
Buttafuoco, 2024	Y	Y	Y	Y	Y	Y	Y	Y	Y	Y	Y
Callahan, 2017	Y	N	Y	Y	N	Y	Y	Y	N	Y	Y
Campbell, 2020	Y	Y	Y	Y	Y	Y	N	Y	Y	Y	Y
Capito, 2025	Y	Y	Y	Y	Y	Y	Y	P	N	Y	Y
Ceusters, 2018	Y	Y	Y	Y	Y	Y	Y	N	Y	Y	Y
Chehal, 2025	Y	Y	Y	Y	Y	P	Y	N	Y	Y	Y
Chen, 2016	Y	Y	Y	Y	U	Y	N	Y	N	Y	Y
Chen, 2024	Y	Y	Y	Y	Y	Y	Y	Y	N	Y	Y
Cillessen, 2020	Y	Y	Y	Y	Y	P	Y	N	Y	P	Y
Cillessen, 2021	Y	Y	Y	Y	Y	Y	Y	Y	N	Y	Y
Cohen, 2019	Y	Y	Y	Y	N	Y	Y	Y	Y	Y	Y
Cyr, 2016	Y	Y	Y	N	Y	Y	Y	Y	N	Y	Y
Daskivich, 2018	Y	Y	Y	Y	Y	Y	Y	Y	N	Y	Y
Devarakonda, 2017	Y	Y	Y	Y	Y	Y	Y	Y	Y	Y	Y
Doghramji, 2018	Y	Y	Y	Y	Y	Y	Y	Y	N	Y	Y
Durojaiye, 2018	Y	Y	Y	Y	Y	Y	Y	Y	Y	Y	Y
Flanagan, 2016	Y	Y	Y	Y	Y	Y	Y	Y	Y	Y	Y
Gammal, 2021	Y	Y	Y	Y	Y	Y	Y	Y	Y	Y	Y
Grauer, 2022	Y	Y	Y	Y	Y	Y	Y	Y	Y	Y	Y
Harris, 2020	Y	Y	Y	Y	Y	Y	Y	Y	Y	Y	Y
Heintzman, 2020	Y	Y	Y	Y	Y	Y	Y	Y	Y	Y	Y
Hier, 2019	Y	Y	Y	Y	Y	Y	Y	Y	Y	Y	Y
Hodge, 2020	Y	Y	Y	Y	Y	Y	Y	Y	Y	Y	Y
Horng, 2019	Y	Y	Y	Y	Y	Y	Y	Y	Y	Y	Y
Hose, 2019	Y	Y	Y	Y	Y	Y	Y	Y	Y	Y	Y

Idres, 2025	Y	Y	Y	Y	Y	Y	Y	Y	U	Y	Y
Jackson, 2023	Y	Y	Y	Y	Y	Y	Y	Y	Y	Y	Y
Jiggins, 2016	Y	N	Y	Y	Y	Y	Y	Y	Y	Y	Y
Joseph, 2016	Y	Y	Y	Y	Y	Y	Y	Y	Y	Y	Y
Kagan, 2017	Y	Y	Y	Y	Y	Y	Y	Y	Y	Y	Y
Kieft, 2017	Y	Y	Y	Y	Y	Y	Y	Y	Y	Y	Y
King, 2022	Y	Y	Y	Y	Y	Y	Y	Y	Y	Y	Y
Klappe, 2020	Y	Y	Y	Y	Y	Y	Y	Y	Y	Y	Y
Klappe, 2021	Y	Y	Y	Y	Y	Y	Y	Y	Y	Y	Y
Klappe, 2023	Y	Y	Y	Y	Y	Y	Y	Y	Y	Y	Y
Klein, 2025	Y	Y	Y	Y	Y	Y	Y	Y	Y	Y	Y
Krauss, 2016	Y	Y	Y	Y	Y	Y	Y	Y	Y	Y	Y
Kreuzthaler, 2019	Y	Y	Y	Y	Y	Y	Y	Y	Y	Y	Y
Kreuzthaler, 2024	Y	Y	Y	Y	Y	Y	Y	Y	Y	Y	Y
Li, 2018	Y	Y	Y	Y	Y	Y	Y	Y	Y	Y	Y
Li, 2020	Y	Y	Y	Y	Y	Y	Y	Y	Y	Y	Y
Liang, 2017	Y	Y	Y	Y	Y	Y	Y	Y	Y	Y	Y
Liang, 2021	Y	Y	Y	Y	Y	Y	Y	Y	Y	Y	Y
Liao, 2020	Y	Y	Y	Y	Y	Y	Y	Y	Y	Y	Y
Mangin, 2020	Y	N	Y	Y	U	Y	Y	Y	Y	Y	Y
Marquez Fosser, 2017	Y	Y	Y	Y	Y	Y	Y	Y	Y	Y	Y
Martin, 2020	Y	Y	Y	Y	Y	Y	Y	Y	Y	Y	Y
Martin, 2021	Y	Y	Y	Y	Y	Y	Y	Y	Y	N	Y
Mattar, 2017	Y	Y	Y	Y	Y	Y	Y	Y	Y	N	Y
McEvoy, 2018	Y	Y	Y	Y	Y	Y	Y	Y	Y	Y	Y
McNeely, 2025	Y	Y	Y	Y	Y	Y	Y	Y	Y	Y	Y
Meredith, 2020	Y	Y	Y	Y	Y	Y	Y	Y	Y	Y	Y
Meredith, 2021	Y	Y	Y	Y	Y	Y	Y	Y	Y	Y	Y
Meyerhoefer, 2017	Y	Y	Y	Y	Y	Y	Y	Y	Y	Y	Y
Molina, 2025	Y	Y	Y	Y	Y	Y	Y	Y	Y	Y	Y
Nasir, 2018	Y	Y	Y	Y	Y	Y	Y	Y	Y	Y	Y
Nelson, 2018	Y	Y	Y	Y	Y	Y	Y	Y	Y	Y	Y
Nugent, 2025	Y	Y	Y	Y	Y	Y	Y	Y	Y	Y	Y
Pahl, 2016	Y	Y	Y	Y	Y	Y	Y	Y	Y	Y	Y
Paridaens, 2026	Y	Y	Y	Y	Y	Y	Y	Y	Y	Y	Y
Patel, 2016	Y	Y	Y	Y	Y	Y	Y	Y	Y	Y	Y
Porter, 2020	Y	Y	Y	Y	Y	Y	Y	Y	Y	Y	Y
Poulos, 2021	Y	Y	Y	Y	Y	Y	Y	Y	Y	Y	Y
Prater, 2019	Y	Y	Y	Y	Y	Y	Y	Y	Y	Y	Y

Prazeres, 2025	Y	Y	Y	Y	Y	Y	Y	Y	Y	Y	Y
Rajbhandari, 2018	Y	Y	Y	Y	Y	Y	Y	Y	Y	Y	Y
Ridgway, 2021	Y	Y	Y	Y	Y	Y	Y	Y	Y	Y	Y
Rodriguez, 2017	Y	Y	Y	Y	Y	Y	Y	Y	Y	Y	Y
Sandhu, 2024	Y	Y	Y	Y	Y	Y	Y	Y	Y	Y	Y
Sanford, 2021	Y	Y	Y	Y	Y	Y	Y	Y	Y	N	Y
Satti, 2021	Y	Y	Y	Y	Y	Y	Y	Y	Y	Y	Y
Sauer, 2024	Y	Y	Y	Y	Y	P	Y	Y	Y	Y	Y
Senior, 2024	Y	Y	Y	Y	Y	Y	Y	Y	Y	Y	Y
Simon, 2025a	Y	Y	Y	Y	Y	Y	Y	Y	Y	Y	Y
Simon, 2025b	Y	Y	P	P	Y	Y	Y	Y	Y	Y	Y
Singer, 2016	Y	Y	Y	Y	Y	Y	Y	Y	Y	Y	Y
Singer, 2017	Y	Y	Y	Y	Y	Y	Y	Y	Y	Y	Y
Sinha, 2017	Y	Y	Y	Y	Y	Y	Y	Y	Y	Y	Y
Smith, 2021	Y	Y	Y	Y	Y	Y	Y	Y	Y	Y	Y
Smits, 2016	Y	N	Y	Y	Y	Y	Y	Y	Y	Y	Y
Sockolow, 2019	Y	Y	Y	Y	Y	Y	Y	Y	Y	Y	Y
Sockolow, 2021	Y	Y	Y	Y	Y	Y	Y	Y	Y	Y	Y
Stein, 2019	Y	Y	Y	Y	Y	Y	Y	Y	Y	Y	Y
Sutton, 2019	Y	Y	Y	N	Y	Y	Y	Y	Y	Y	Y
Tan, 2024	Y	Y	Y	Y	Y	Y	Y	Y	Y	Y	Y
Teeple, 2023	Y	Y	Y	Y	Y	Y	Y	Y	Y	Y	Y
Tomita, 2019	Y	Y	Y	Y	Y	Y	Y	Y	Y	Y	Y
Vera Ramos, 2019	Y	Y	Y	Y	Y	Y	Y	Y	Y	Y	Y
Vivthcarenko, 2021	Y	Y	Y	Y	Y	Y	Y	Y	Y	Y	Y
Voss, 2022	Y	Y	Y	Y	Y	Y	Y	N	Y	Y	Y
Wang, 2019	Y	Y	Y	Y	Y	Y	Y	N	Y	Y	Y
Wang, 2020	Y	Y	Y	Y	Y	Y	Y	Y	Y	Y	Y
Wang, 2021	Y	Y	Y	Y	Y	Y	Y	Y	Y	Y	Y
Wardell, 2025	Y	Y	Y	Y	Y	Y	Y	Y	P	Y	Y
Weiskopf, 2019	Y	Y	Y	Y	Y	Y	Y	Y	Y	Y	Y
Wright, 2023	Y	Y	Y	Y	Y	Y	Y	Y	N	Y	Y
Xu, 2018	Y	Y	Y	Y	Y	Y	Y	Y	Y	Y	Y
Zahar, 2018	Y	Y	Y	Y	Y	Y	Y	Y	N	Y	Y

* Critical appraisal was conducted using a modified JBI checklist adapted for rapid scoping reviews and heterogeneous study designs. Checklist items were scored as Yes (criterion clearly met), No (criterion not met), Not Applicable (NA; criterion not relevant to the study design), or Unclear (U; insufficient methodological detail to permit judgement). Consistent with PRISMA-ScR guidance, appraisal results were used to support interpretation of the evidence base and not to exclude studies. The checklist was adapted for descriptive purposes only.
